# *Toxocara* Seroprevalence in Patients with Idiopathic Parkinson's Disease: Chance Association or Coincidence?

**DOI:** 10.1155/2013/685196

**Published:** 2012-12-26

**Authors:** Tuncay Çelik, Yüksel Kaplan, Eser Ataş, Derya Öztuna, Said Berilgen

**Affiliations:** ^1^School of Health, Adiyaman University, 02040 Adiyaman, Turkey; ^2^Department of Neurology, Faculty of Medicine, Inonu University, 44280 Malatya, Turkey; ^3^Department of Neurology, Faculty of Medicine, Firat University, 23119 Elazig, Turkey; ^4^Department of Biostatistics, Faculty of Medicine, Ankara University, 06100 Ankara, Turkey

## Abstract

Most cases of idiopathic Parkinson disease (IPD) are believed to be due to a combination of genetic and environmental factors. The purpose of this study is to investigate the relationship between toxocariasis and Parkinson disease (PD). Patients were selected from people who were admitted to the Movement Disorders Branch, Neurology Department of Elazığ University Faculty of Medicine Elazığ, Turkey. We studied specific IgG antibodies against *Toxocara canis* (*T. canis*) in 50 patients with idiopathic Parkinson and 50 healthy volunteers. We investigated the clinical history of three patients infected with *T. canis*. We also studied specific IgG antibodies against *Toxoplasma gondii* in these groups. Antibodies anti-*Toxocara canis* were found in 3 idiopathic PD (6%) (*P* = 0.121) and antibody titer was not found in control. A patient had history of the presence of dog in current dog ownership. We did not detect any statistically significant association between *T. canis* and IPD. But, we believe that further comprehensive studies are required for understanding whether there is a causal relation between toxocariasis and PD. We didn't find possible association between *Toxoplasma gondii* and IPD (*P* = 0.617).

## 1. Introduction

 The idiopathic form of Parkinson disease (IPD) is one of the most common neurodegenerative disorders. It is mainly characterized by a progressive and massive loss of dopaminergic (DA) neurons in the substantia nigra *pars compacta* (SNpc), which leads to several clinical motor symptoms such as akinesia, rigidity, and resting tremor. The molecular pathways leading to these concomitant clinical alterations remain obscure, but it is believed that it may result from environmental factors, genetic causes, or a combination of the two [1]. The frequency of contact with infection agents in patients with IPD and the relationship between infections and clinical findings or pathogenesis of IPD are unclear. In relation to this, a few studies in the literature have drawn attention to the possible role of infectious agents in the pathogenesis of IPD [2–5].

 Toxocariasis is caused by ingestion of the eggs of *Toxocara canis *(*T. canis*), a roundworm in dogs, puppies, and cats [6]. Human who ingest *T. canis* eggs may remain unaffected or may manifest a mono- or multisystem disease with symptoms arising from larval invasion of different organs. Though neurological manifestations of *T. canis* larvae are rare, toxocariasis remains an important differential diagnosis of various neurological disorders [6, 7]. Neurological manifestations of toxocariasis have documented both the central and the peripheral nervous system. Dementia, behavioral disturbances, meningoencephalitis, myelitis, cerebral vasculitis, epilepsy, optic neuritis, radiculitis, affection of cranial nerves, lower motor neuron disease, and musculoskletal involvement have all been associated with neurotoxocariasis [6–12]. However, a possible relationship between *T. canis* infection and IPD has not been reported in the literature, but a few studies suggested a possible change in the neurotransmitter levels such as GABA, dopamine, serotonin, and monoamines in *Toxocara*-infected animals [13, 14]. This observation drew our attention to a possible link between PD and *T. canis* infection. In order to further explore this association, we evaluated the *Toxocara* antibodies in patient with IPD and compared the results with those of control group.

## 2. Materials and Methods

### 2.1. Patients and Evaluation

The patients were diagnosed with IPD according to the United Kingdom Parkinson's Disease Society Brain Bank Clinical Diagnostic Criteria in the Neurology Outpatient Clinic of the Medical Faculty of Elazığ University. All patients were randomly selected from the database of the neurology department. Control group were randomly selected among the healthy people who underwent a complete neurological examination to exclude the presence of neurological disorders and were matched to the case patients by age and gender. Demographic characteristics and information concerning factors possibly associated with *T. canis* infection were evaluated by a structured interview. We recorded data as follows: age, gender, duration of IPD, the presence of dog in the house, and clinic symptoms (dementia, epilepsy headache, fever, chronic urticaria, etc.). The study was performed in accordance with the Helsinki Declaration. All subjects signed informed consent after receiving a detailed explanation of the study procedures.

### 2.2. Serological Detection of *Toxocara* Canis Infection

Five milliliters blood samples were obtained from the cubital vein of both the patients and controls. The sera were separated from whole blood shortly after collection and was stored at −20°C until the analysis. The sera were examined by ELISA *T*. *canis* IgG test (NOVATEC, Immundiagnostica GmbH). In the next step, in order to detect possible involvement of *Toxoplasma gondii* in patients, the sera were also examined by ELISA *Toxoplasma gondii* IgG test (Cobas Core, Roche, Germany).

### 2.3. Statistical Assessment

Statistical evaluations were performed by using the SPSS 11.5 programme. For metric variables mean ± standard deviation (minimum-maximum), for categorical variables frequency (percentage) was used as descriptive statistics. Student *t*-test was used to compare mean ages of groups. chi-square test was used to determine whether there was a difference between groups in terms of *T. canis* positivity. *P* < 0.05 was considered as statistically significant. 

## 3. Results

Fifty patients with IPD and fifty controls were enrolled in the study. The mean age of patients was 65.6 ± 10.2 (range: 43–81). Of the 50 patients, 32 (64%) were males and 18 (36%) were females. The mean duration of IPD was 5 ± 4 years (range: 1–17). The mean age of controls was (65.6 ± 30.4). Among the 50 controls, 29 (58%) were males and 21 (32%) were females. There were no statistically significant differences among the patients and controls subjects with respect to age and gender (*P* = 1.000 and 0.818, resp.). While 3 out of 50 (6%) cases with IPD were positive for anti-*T*. *canis* IgG antibodies, all control subjects were negative. There were also no statistically significant differences between the rates of positivity between the IPD patient group and the control group (*P* = 0.121). In 9 (18%) of the 50 patients, and in 11 (22%) of 50 control group, anti-*Toxoplasma gondii* antibodies were detected (*P* = 0.617). Sociodemographic characteristics and clinical characteristics among groups are given in Table 1.

### 3.1. Presentation of the Patients with Positive *Toxocara* Antibodies

**Patient 1**

A 78-year-old male had a ten-year history of IPD. He had no history of presence of dog in the house and clinic symptoms of toxocariasis.

**Patient 2**

A 46-year-old male reported a history of IPD for two years. He had a dog in the house. But the patient reported no clinic symptoms of toxocariasis.

**Patient 3**

A 76-year-old male had an eight-year history of IPD. He had neither the presence of dog in the house nor clinic symptoms of toxocariasis.

*Toxoplasma gondii* IgG antibodies were negative in all patients.

## 4. Discussion

In this study, we investigated the *T*. *canis* IgG antibodies in patients diagnosed with IPD. Although *Toxocara* IgG antibodies were found in 3 IPD (6%), all control subjects were negative. IPD is an age-related neurodegenerative disorder that is characterized by a slow and progressive degeneration of DA neurons in the SNpc combined with intracytoplasmic proteinaceous inclusions known as Lewy bodies. Despite an extensive research, the exact cause of IPD is not completely known, but important clues point at the involvement of some combination of genetic and environmental factors [5]. Several potential the pathogenic mechanisms of neuronal degeneration in IPD have been shown, including oxidative stress, neuroinflammatory processes, mitochondrial abnormalities, excitatory amino acids, a rise in intracytoplasmic free calcium, cytokines, and apoptotic processes. Recent studies have revealed an essential role for neuroinflammation that is initiated and driven by activated microglial and infiltrated peripheral immune cells and their neurotoxic products (such as proinflammatory cytokines, reactive oxygen species, and nitric oxide) in the pathogenesis of IPD [15]. Epidemiologic studies suggest that a number of environmental factors may increase the risk of developing IPD. These include exposure to well water drinking, farming, pesticides exposure (paraquat, organophosphates, and rotenone), herbicides, metals (manganese, copper, mercury, lead, iron, zinc, and aluminum), wood pulp mills, rural residence, diet, head trauma, and infections [5, 15]. Parkinsonian states have been described following viral encephalitis due to measles, Japanese B virus, Western equine virus, polio, epstein-barr virus, cytomegalovirus, influenza A virus, and human immunodeficiency virus disease [5].

More recently, Miman et al. found *Toxoplasma gondii* IgG antibodies in IPD higher than controls. They speculated that there might be a possible association between *Toxoplasma gondii *and IPD and *Toxoplasma* infection may be involved in the pathogenic mechanisms of IPD [4]. But, our both studies did not detect any statistically significant association between *T. gondii* and PD [3].

The clinical presentations of toxocariasis have been documented both the central nervous system and the peripheral nervous system in the literature since 1951. More recently, the biochemical and immunopathological alterations in the brain in experimental neurotoxocariasis model have also been shown [13, 16]. Othman et al. demonstrated significant increase in gene expression of proinflammatory cytokines and nitric oxide in the brains of *Toxocara*-infected mice especially in the chronic stage. Proinflammatory cytokines can be neuroprotective, but with sustained or overstimulated increase, they will have deleterious effects on the neurons [14]. They found also significant changes in neurotransmitter profile. They reported that *T. canis* infected mice demonstrated significantly decreased GABA, dopamine, monoamines, and serotonin levels compared with uninfected mice. However, norepinephrine levels were higher in infected mice in this study.

We could not find any published study which has evaluated the seroprevelance of toxocariasis in IPD patients. Besides, there are a few studies in Turkey about seroprevalence *Toxocara*. These studies show that the seroprevalence of toxocariasis varies between 2.6% and 51% in our country [17–23]. All of these studies except two reports have been done in childhood. One of these studies, Kaplan et al. showed that *T. canis *seroprevalence was 2.6% in healthy individuals in Elazığ, an urban region in Turkey [17]. Another study has shown that the seroprevalence was 6% in students at veterinary college and 10% in people exposed to dogs [18].

We found seroprevelance of *Toxocara* as 7.1% in patients with IPD and 0% in the controls. Despite the high anti-*T. canis* seropositivity found in our IPD patients, we did not detect any statistically significant differences between the rates of positivity between the IPD patient group and the control group. The relationship between anti-*T. canis* seropositivity and IPD may be chance association in these cases. On the other hand, the rate of anti-*T. canis* seropositivity in our patients increased nearly by a threefold when compared with study performed from Elazığ in healthy individuals. Furthermore, experimental models of neurotoxocariasis have also shown increased neuroinflammatory mechanisms and significant changes in neurotransmitter profile which all of neurotransmitters also play mainly role in IPD. 

## 5. Conclusion

The present study suggest the possibility that the presence of the *Toxocara* IgG antibodies in patients diagnosed with IPD may extend beyond the chance. The pathogenic mechanisms involved in IPD remain incompletely understood. Based on the concept that IPD seems to be a multifactorial disease, genetic factors, oxidative stress, and neuroinflammatory processes have been implicated in its pathogenesis [24]. We believe that further comprehensive studies are required for understanding whether there is a causal relation between toxocariasis and IPD and/or the presence of the *Toxocara* IgG antibodies may be the possibility risk factor for IPD.

## Figures and Tables

**Table 1:** Comparison of demographic and clinical characteristics between groups.

Parameters	Patients (*n*: 50)	Controls (*n*: 50)	*P*
Gender			
Male	35 (50.7)	34 (49.3)	*P* = 0.824
Female	15 (48.4)	16 (51.6)	
Ages (years)	75.0 ± 73.92	64.4 ± 10.61	*P* = 0.21
Duration of IPD (years)	4.73 ± 3.57 (min: 1–max: 17)	—	—
Positive for* Toxocara canis* IgG antibodies	3 (6%)	0 (0%)	*P* = 0.121
Positive for* Toxoplasma gondii* IgG antibodies	9 (18.0%)	11 (22.0%)	*P* = 0.617
